# Latrophilins and Teneurins in Invertebrates: No Love for Each Other?

**DOI:** 10.3389/fnins.2019.00154

**Published:** 2019-03-12

**Authors:** Torsten Schöneberg, Simone Prömel

**Affiliations:** Medical Faculty, Rudolf Schönheimer Institute of Biochemistry, Leipzig University, Leipzig, Germany

**Keywords:** adhesion GPCRs, Latrophilins, Teneurins, invertebrates, interaction

## Abstract

Transsynaptic connections enabling cell–cell adhesion and cellular communication are a vital part of synapse formation, maintenance and function. A recently discovered interaction between the Adhesion GPCRs Latrophilins and the type II single transmembrane proteins Teneurins at mammalian synapses is vital for synapse formation and dendrite branching. While the understanding of the effects and the molecular interplay of this Latrophilin-Teneurin partnership is not entirely understood, its significance is highlighted by behavioral and neurological phenotypes in various animal models. As both groups of molecules, Latrophilins and Teneurins, are generally highly conserved, have overlapping expression and often similar functions across phyla, it can be speculated that this interaction, which has been proven essential in mammalian systems, also occurs in invertebrates to control shaping of synapses. Knowledge of the generality of this interaction is especially of interest due to its possible involvement in neuropathologies. Further, several invertebrates serve as model organisms for addressing various neurobiological research questions. So far, an interaction of Latrophilins and Teneurins has not been observed in invertebrates, but our knowledge on both groups of molecules is by far not complete. In this review, we give an overview on existing experimental evidence arguing for as well as against a potential Latrophilin-Teneurin interaction beyond mammals. By combining these insights with evolutionary aspects on each of the interaction partners we provide and discuss a comprehensive picture on the functions of both molecules in invertebrates and the likeliness of an evolutionary conservation of their interaction.

## Latophilins and Teneurins Form a Transsynaptic Complex in Mammals

The formation of synapses is one of the key steps in warranting the development of a functioning neuronal network. This highly complex process is not fully understood, but it involves various interactions of molecules with adhesive and transmembrane signaling properties. A pair of proteins which has recently taken the stage to be essential for synaptic organization in many vertebrates are Latrophilins and Teneurins. Both have already been separately recognized as synaptic cell surface proteins several decades ago.

**FIGURE 1:**
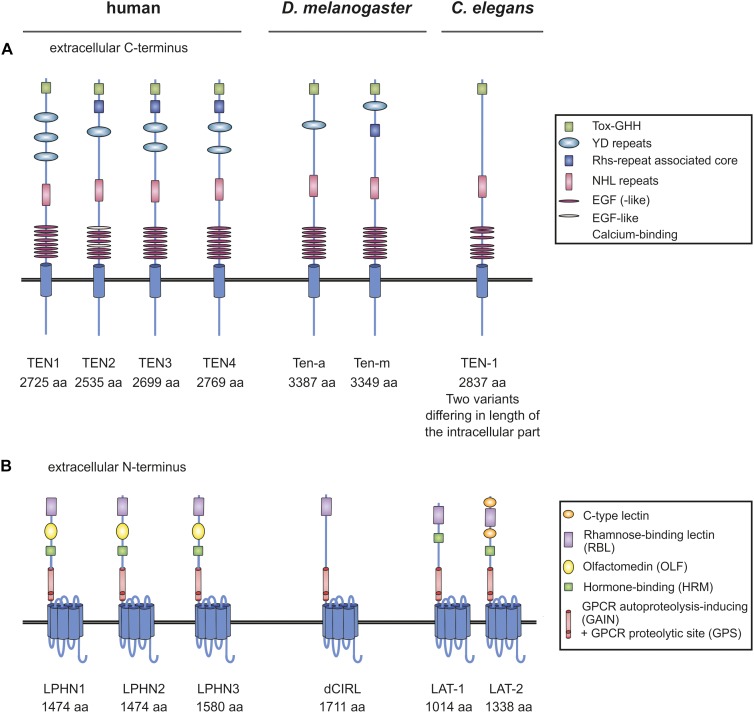
Organization of Teneurins and Latrophilins in vertebrates and invertebrates. **(A)** Schematic depiction of Teneurins in human, fruit fly and worm. YD repeats are indicated as regions, not as single repeats. The function of many of the domains remains elusive. **(B)** Domain architecture of Latrophilins. Due to several splice variants of the Teneurin and Latrophilin homologs not every variant is depicted, but only the longest one. Note that all receptor molecules are not drawn to scale. Domains were annotated using InterPro (EMBL-EBI) and SMART (Letunic et al., 2009).

Teneurins are large type II one-transmembrane domain proteins with a cytoplasmic N-terminus and an extracellularly located C-terminus containing tyrosine-aspartate (YD) repeats and numerous epidermal growth factor (EGF) domains (Oohashi et al., 1999; Tucker and Chiquet-Ehrismann, 2006; Figure 1A). They have various neuronal functions, for example in mediating interneuronal connections, promoting synapse formation and shaping dendritic morphology in diverse types of neurons in vertebrates and invertebrates (Hong et al., 2012; Mosca et al., 2012; Antinucci et al., 2013; Berns et al., 2018). Consistently, the four vertebrate homologs (TEN1–4) are widely expressed in the developing and the adult brain, for instance in the hippocampus, the cerebellum and the visual cortex (Oohashi et al., 1999; Tucker et al., 2000; Rubin et al., 2002; Zhou et al., 2003; Kenzelmann et al., 2008). Studies on animal models further reveal the essential impact of Teneurins on neuronal circuits. For example, mice knockout for *Ten3* display neurological defects, in particular deficits in visually mediated behavior (Leamey et al., 2007). Similarly, in zebrafish, knockdown of Ten-3 leads to retinal ganglion cell stratification defects (Antinucci et al., 2013).

The molecular details underlying Teneurin function involve the formation of homotypic or heterotypic dimers depending on the synapse type [summarized in Mosca (2015)]. Most details on Teneurin function, however, have not been collected in vertebrates, but using the fruit fly *Drosophila melanogaster* as a model (section “Latrophilins and Teneurins in *D. melanogaster*–No Evidence for Interaction”).

The functions of Latrophilins have by far not been as well characterized as the ones of Teneurins. Latrophilins belong to the class of Adhesion G protein-coupled receptors (Adhesion GPCRs, aGPCRs). The three mammalian homologs (LPHN1–3/ADGRL1-3) comprise an intracellular C-terminus, a seven transmembrane region (7TM) and an extracellular N-terminus containing a rhamnose-binding lectin (RBL), an olfactomedin (OLF), a hormone binding (HRM) and a GPCR autoproteolysis-inducing (GAIN) domain, which harbors the GPCR proteolytic site (GPS) (Figure 1B). Latrophilins first came into the focus of science as targets of α-Latrotoxin, a component of the Black widow spider’s toxin (Krasnoperov et al., 1996; Lelianova et al., 1997; Sugita et al., 1998). Specifically, Latrophilin-1 (LPHN1/ADGRL1) has subsequently been characterized to be expressed in various neurons of the murine central nervous system and evidence exists that LPHN1 is localized presynaptically (Silva et al., 2011; Vysokov et al., 2018) as well as on the post-synapse (Tobaben et al., 2000; Anderson et al., 2017). The impact of this localization on both sides of the synapse has not been clarified to date. Studies on LPHN3 in mouse and zebrafish models suggest a role for the receptor in the dopaminergic system and an association of variants in the receptor gene with the pathogenesis of attention-deficient hyperactive disorder (ADHD) (Arcos-Burgos et al., 2010; Lange et al., 2012; Wallis et al., 2012).

**FIGURE 2:**
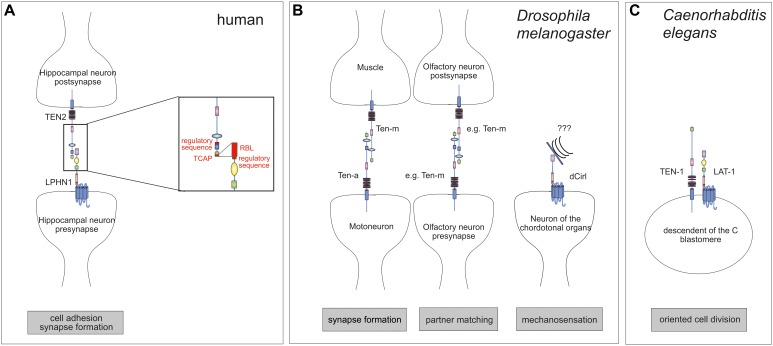
Interaction of Teneurins and Latrophilins. **(A)** In mammals, the interaction between LPHN1 on the pre-synapse of hippocampal neurons with postsynaptic TEN2 contributes to the control of synapse formation. The interaction interfaces are roughly known. TEN2 binds to LPHN1 via the C-terminal portion of the Tox-GHH domain, the Teneurin C-terminal-associated peptide (TCAP). A short amino acid sequence further N-terminal is involved in regulation of the binding. On the LPHN1 side the rhamnose-binding lectin (RBL) is required for binding as well as a sequence between the RBL and olfactomedin (OLF) domains. **(B)** In the fruit fly, the Teneurins Ten-a and Ten-m interact heterophilically at the neuromuscular junction to ensure synapse formation. Further, a homophilic interaction between Teneurins controls partner matching for instance in the olfactory system. The only Latrophilin homolog in *Drosophila*, dCirl, is located on neurons of the chordotonal organs and are involved in mechanosensation. **(C)** The *C. elegans* homologs of Latrophilins, LAT-1, and Teneurin, TEN-1, are present on the same embryonic blastomeres, excluding the possibility of a classical ligand-receptor pair. Rather, they are acting in parallel. Note that it is rather likely that for any of the interactions shown additional molecules or dimerization are required which are not depicted here.

Teneurins and Latrophilins are both found enriched in neuronal growth cones (Nozumi et al., 2009). Recently, strong evidence has been provided that mammalian Teneurins and Latrophilins form heterophilic dimers at the synapse. This interaction, which occurs between LPHN1 and the Teneurin homolog TEN2 [also termed Lasso (Silva et al., 2011)], is transsynaptic and mediates cell adhesion (Silva et al., 2011; Boucard et al., 2014; Li et al., 2018). As a consequence, it induces synapse formation in murine hippocampal neurons and neuronal cultures (Silva et al., 2011; Figure 2A). Another study has shown that besides TEN2 also TEN4, but not TEN1 is able to bind LPHN1 (Boucard et al., 2014). It needs to be noted that not for all Teneurin functions in neurons, interaction with Latrophilin is essential [reviewed in Mosca (2015)]. It has only proven vital for cell adhesion and synapse formation so far. For its other roles homophilic interactions or different heterophilic partners have been shown.

Although the molecular details of the interaction between Latrophilins and Teneurins have not been clarified yet, the regions within both molecules taking part in the intermolecular interaction have been roughly identified (Figure 2A) using binding assays and mutation analyses. For TEN2, the interaction is mediated via its C-terminal portion, mainly by a sequence within the Tox-GHH domain, the so-called Teneurin C-terminal-associated peptide (TCAP). This sequence can act as a bioactive peptide upon cleavage and shapes dendritic morphology, stimulates neurite outgrowth and mediates anxiety behavior (Wang et al., 2005; Al Chawaf et al., 2007a,b; Tan et al., 2011). Interestingly, besides this core sequence within the Tox-GHH domain, a 7-amino acid-long region located in a β-propeller close to the NHL (NCL-1/HT2A/Lin-41) repeats also regulates binding (Li et al., 2018). The same seems to be true for the interaction site within Latrophilins. While the presence of the RBL domain is mainly responsible for binding Teneurins (Boucard et al., 2014), an alternative exon encoding a region between RBL and OLF domains modulates binding affinity to TEN2 (Boucard et al., 2014). It needs to be noted that currently existing data on the partnership of Latrophilins and Teneurins does not exclude the possibility that the interaction occurs in the context of a larger complex involving other molecules. This scenario has been already proposed (Woelfle et al., 2015, 2016) based on the findings that Teneurin also interact with dystroglycans (Chand et al., 2012) and Latrophilins bind to Neurexins (Boucard et al., 2012) or (in a complex) to fibronectin leucine-rich transmembrane (FLRT) proteins (O’Sullivan et al., 2012, 2014; Jackson et al., 2015; Lu et al., 2015). These interaction partners are all expressed by neurons.

As both, Latrophilins and Teneurins, can act as ligand, it is conceivable that each of them functions as receptor transducing signals into their host cell. It has not been determined beyond doubt to date which of them is the ligand and which the signal-receiving molecule or if both of them signal. However, some studies show that Teneurins are cleaved at several distinct sites rendering liberated fragments (Wang et al., 2005), which are involved in different functions in the brain such as neurite outgrowth (Al Chawaf et al., 2007a; Erb et al., 2014). It has been suggested that one of these TEN2 fragments, generated by regulated proteolysis, is soluble and can still bind LPHN1 and trigger signaling (Silva et al., 2011; Vysokov et al., 2016, 2018) indicating that LPHN1 is the receptor transducing information into the cell.

## Latrophilins and Teneurins in Invertebrates Have Similar Functions

Due to the obvious relevance of the Latrophilin-Teneurin interaction in mammals the question of the generality of this partnership and thus, its conservation, arises. This question is especially of interest as invertebrate models are often used for elucidation of neurobiological aspects and understanding of association with pathologies. The described interaction between Latrophilins and Teneurins is so far limited to vertebrates, it has not been shown in invertebrate systems to date. However, Teneurins and Latrophilins are both highly conserved groups of molecules. First discovered in the fruit fly *Drosophila melanogaster* (Baumgartner and Chiquet-Ehrismann, 1993; Levine et al., 1994), Teneurins are evolutionarily as old as the unicellular choanoflagellates and are present in all metazoa investigated so far (Tucker and Chiquet-Ehrismann, 2006; Tucker et al., 2012). Similarly, Latrophilins belong to the evolutionarily oldest groups of Adhesion GPCRs being present in vertebrates and in invertebrates. Functionally, Teneurins also seem to be highly conserved, not only in respect to their neuronal roles (sections “Latrophilins and Teneurins in *D. melanogaster*–No Evidence for Interaction” and “Latrophilins and Teneurins in *C. elegans* Development Do Not Function as Ligand-Receptor Pair”). The receptors also have functions beyond synapse formation. It has been shown in mice that TEN4 is required for mesoderm induction and gastrulation (Lossie et al., 2005; Nakamura et al., 2013). Consistently, non-neuronal expression of mammalian Teneurins is found during embryonic development. This pattern is similar to the one of the *Caenorhabditis elegans* ortholog, suggesting conserved non-neuronal functions (section “Latrophilins and Teneurins in *C. elegans* Development Do Not Function as Ligand-Receptor Pair”). In contrast, for all that is known to date, the functional conservation of Latrophilins throughout phyla has not been shown beyond doubt (sections “Latrophilins and Teneurins in *D. melanogaster*–No Evidence for Interaction” and “Latrophilins and Teneurins in *C. elegans* Development Do Not Function as Ligand-Receptor Pair”).

Due to the overall similar conservation of the two molecules it has been postulated that their interaction and its physiological impact are also evolutionarily old and conserved (Chand et al., 2013; Woelfle et al., 2015). Although experimental proof is lacking that in invertebrates Latrophilins and Teneurins interact, a body of functional proof in the invertebrate model organisms *D. melanogaster* and *C. elegans* exists suggesting that an interaction of the two is conceivable. However, there is also some information arguing against this assumption which will be discussed below.

### Latrophilins and Teneurins in *D. melanogaster* – No Evidence for Interaction

Teneurins were first discovered in the fruit fly *D. melanogaster* as pair-rule genes tenascin-like molecule accessory (Ten-a) (Baumgartner and Chiquet-Ehrismann, 1993) and tenascin-like molecule major (Ten-m) (Baumgartner et al., 1994), which was also named odd oz (Odz) (Levine et al., 1994). The structural (Figure 1A) and functional conservation between mammalian and *Drosophila* Teneurins is evident. In *Drosophila*, Teneurins are widely expressed in neurons of the central and peripheral nervous system (Minet et al., 1999; Fascetti and Baumgartner, 2002) and several studies show their involvement in two different aspects at the synapse. Firstly, screens have revealed that they contribute to synapse formation of the neuromuscular junction (Liebl et al., 2006; Kurusu et al., 2008; Figure 2B). Further, Teneurins have implications in partner matching between presynaptic motoneurons and postsynaptic muscles as well as pre- and postsynaptic olfactory neurons and pre-synaptic motoneurons with postsynaptic muscles (Figure 2B; Hong et al., 2012; Mosca et al., 2012). Both functions can also be discriminated based on the connections that are formed by Teneurins. While in synaptogenesis Teneurins interact heterophilically (with another molecule or another Teneurin), they form homophilic interactions (with the same Teneurin) during partner matching. The heterophilic interaction partners described consist of presynaptic Ten-a and postsynaptic Ten-m and deletion of each of the molecules yields dysfunctional synapses and less synaptic boutons (Mosca et al., 2012). It is conceivable that Latrophilin might be another partner for a heterophilic interaction of Teneurins in this context. However, for Latrophilins, the functional conservation between mammals and *Drosophila* is not that evident, which is partly due the lack of knowledge about the receptor in the fruit fly. The one Latrophilin homolog the *Drosophila* genome carries, *dCirl*, has only been recently characterized. It is located on the neuronal dendrites and cilia of chordotonal organs in the fly and mediates sensitivity to touch (Scholz et al., 2015). This Adhesion GPCR is involved in mechanosenation, specifically shaping mechanically gated receptor currents by decreasing intracellular cyclic AMP levels, possibly by activating G_i_ proteins (Scholz et al., 2017). The details of this function remain elusive and thus, no interaction with one of the Teneurins has been described so far. Such interaction can be debated because Ten-m/Odz and dCirl both are located in neurons of the chordotonal organs (Levine et al., 1997), but it seems that they are on the same cell rather than on opposing neurons. However, a partnership might still be likely as we are only just beginning to understand the functions of dCirl.

### Latrophilins and Teneurins in *C. elegans* Development Do Not Function as Ligand-Receptor Pair

In the roundworm *C. elegans*, Teneurins and Latrophilins appear to have very similar functions and at first sight, it can be speculated that they form a classical interaction as described in mammals. However, a closer look prohibits this conclusion as of yet. In contrast to vertebrates or *Drosophila*, the nematode only has one Teneurin gene, *ten-1*. However, several transcript variants exist. The two most prominent ones are generated by two different transcription start sites: one variant with a longer (280 amino acids) intracellular domain and one with a short (36 amino acids) N-terminus (Drabikowski et al., 2005; Figure 1A). Both of these TEN-1 variants are present in distinct subsets of neurons (Drabikowski et al., 2005). Consistently, a role for TEN-1 in neuronal pathfinding has been postulated (Drabikowski et al., 2005). Although *lat-1*, one of the two Latrophilin homologs in *C. elegans* (Figure 1B), is also expressed in neurons (Langenhan et al., 2009), so far no neuronal function of LAT-1 has been described leading to the question whether the classical Latrophilin-Teneurin interaction plays a role in *C. elegans*. LAT-1 is functionally highly diverse. It has roles in fertility and cell polarity during development (Langenhan et al., 2009; Prömel et al., 2012), where it elicits a G_s_ protein-mediated signal raising intracellular cyclic AMP levels (Müller et al., 2015), but a role in synaptogenesis similar to the one in mammals, has not been described yet, precluding a final assessment.

However, similar to mammalian and *Drosophila* Teneurins, expression of *ten-1* is not limited to neuronal tissues but is also found in hypodermal cells (long TEN-1 variant), in cells of the gut, the somatic gonad, distal tip cell, and in few muscle cells (short TEN-1 variant) (Drabikowski et al., 2005). Interestingly, the expression pattern in non-neuronal cells is almost identical to the one of *lat-1*, which is mainly confined to cells of the somatic gonad and the distal tip cell (Langenhan et al., 2009), suggesting that LAT-1 and TEN-1 might have similar functions in a non-neuronal context. Indeed, not only the expression pattern of *lat-1* and *ten-1* is highly similar, but also the phenotype that respective knockout mutants display. Both mutants *lat-1(ok1465)* and *ten-1(ok641)* exhibit morphogenesis defects (Drabikowski et al., 2005; Langenhan et al., 2009). However, genetic analyses revealed that both genes act in parallel during development implying a synergistic rather than linear interaction between *lat-1* and *ten-1* (Langenhan et al., 2009; Figure 2C). In line with these findings, expression data show localization of TEN-1 and LAT-1 on the same embryonic blastomeres rather than on opposing cells, indicating that the two receptors do not form the classical ligand-receptor pair on two different cells in *C. elegans* (Prömel et al., 2012). However, since it is conceivable that Teneurins have multiple functions beyond their role in neurons, it cannot be fully excluded that for some other function, a classical interaction with Latrophilins is required. Further, the second Latrophilin homolog in *C. elegans*, *lat-2*, has not been functionally characterized yet and thus, might also be a candidate for a partnership with TEN-1.

## An Evolutionary View on Latrophilins and Teneurins Points Toward a Young Interaction

Due to their similar expression and function in vertebrates and invertebrates and their high conservation it has been speculated that the Latrophilin-Teneurin interaction also exists in invertebrates (Woelfle et al., 2015). Indeed, a high general sequence conservation of Teneurins from choanoflagellates to vertebrates has been found (Tucker and Chiquet-Ehrismann, 2006) together with structural conservation of core folds and several domains (Jackson et al., 2018; Li et al., 2018). Parts of the Teneurin N-terminus are probably derived from an evolutionarily ancient YD-repeat shell domain that is widespread across the bacterial kingdom by horizontal gene transfer into an early metazoan genome (Jackson et al., 2018). The EGF domains of the Teneurin N-terminus appear first in multicellular animals. Further, comparison of the gene organization among human *Ten1*, *Drosophila* *Ten-a* and *Ten-m* and the *C. elegans* *ten-1* revealed the presence of both, conserved intron locations and exon sequences (Minet and Chiquet-Ehrismann, 2000; Tucker et al., 2012), suggesting that Teneurins arose from a single ancestral gene. This high structural and sequence conservation points toward comparable functions of Teneurins in similar molecular contexts in different species. However, a closer look at the evolution of Latrophilins can cast doubt on the hypothesis that the interaction of Teneurins with Latrophilins is old.

### Invertebrate Latrophilins Are Not One-to-One Orthologs of Mammalian Latrophilins

The class of Adhesion GPCRs belongs to the oldest GPCRs and their sequence signatures in the 7TM part appear first in unicellular organisms such as *Dictyostelium discoideum* and fungi (Krishnan et al., 2012). It needs to be noted that the appearance of genes in unicellular organisms should be taken with caution in the analysis of evolutionary history of gene families due to the possibility of horizontal gene transfer. However, in evolutionarily basal animals such as placozoa (*Trichoplax adhaerens*) and choanoflagellates (*Salpingoeca rosetta* and *Monosiga brevicollis*) there is already a number of Adhesion GPCR-encoding genes indicating their stable integration into animal genomes. Due to high sequence distances it is hard to assign them to Latrophilins or to another of the eight distinct groups of vertebrate Adhesion GPCRs (Nordstrom et al., 2011). Furthermore, none of these evolutionarily old Adhesion GPCRs have been found to present themselves with an RBL-, an OLF-, or an HRM domain (Krishnan et al., 2012), which have been suggested to interact with Teneurins (Woelfle et al., 2015). Therefore, it is rather unlikely that a functional paring of Adhesion GPCRs and Teneurin-like proteins, as described in vertebrates, was already established at this early evolutionary stage, although Teneurin-like proteins are present in placozoa and choanoflagellates.

**FIGURE 3:**
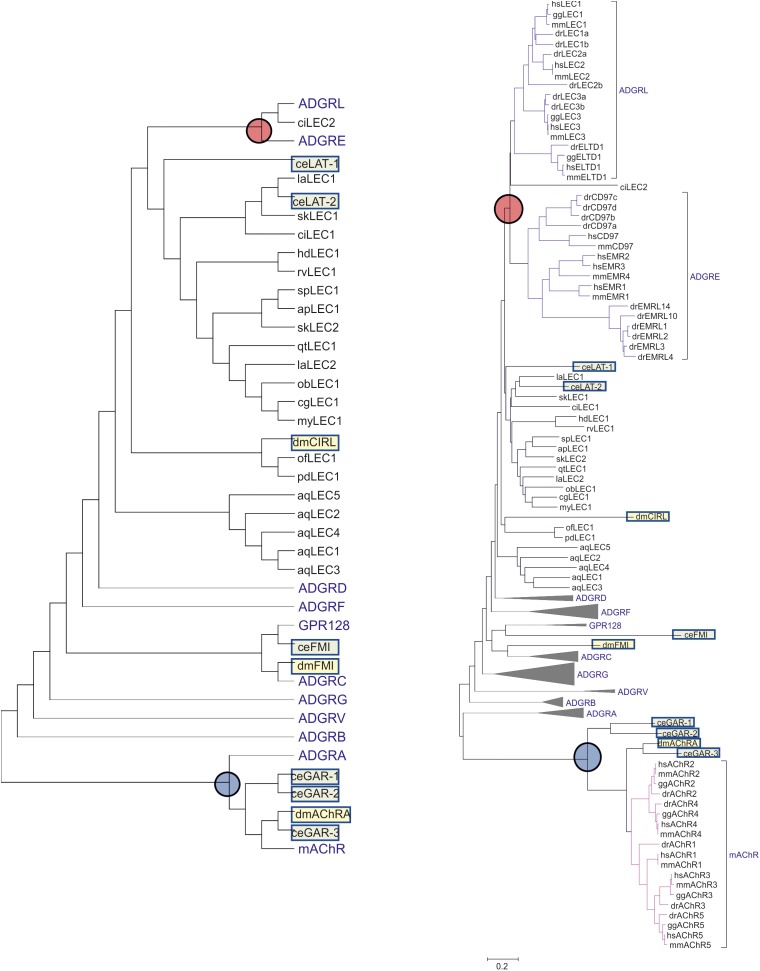
Evolutionary relationships of Latrophilins with other Adhesion GPCR groups. The evolutionary history of *C. elegans* (ce, light green box) and *Drosophila melanogaster* (dm, light yellow box) Latrophilin-like (LAT, Cirl, LEC) and Flamingo-like (FMI) receptors in relation to human, mouse, chicken, and zebrafish Adhesion GPCRs was constructed using the amino acid sequence of the 7TM region. For comparison purposes, Latrophilin-like sequences from other invertebrates were included. Muscarinic acetyl choline receptors of the respective species were used as outgroup. All sequence data are retrieved from NCBI. The evolutionary history was inferred using the Neighbor-Joining method (Saitou and Nei, 1987). The optimal tree with the sum of branch length = 38.4 is shown. The evolutionary distances were computed using the Poisson correction method (Zuckerkandl and Pauling, 1965) and are shown as units of the number of amino acid substitutions per site. The analysis involved 180 amino acid sequences. All positions with less than 95% site coverage were eliminated. That is, fewer than 5% alignment gaps, missing data, and ambiguous bases were allowed at any position. There were a total of 202 positions in the final dataset. Evolutionary analyses were conducted in MEGA7 (Kumar et al., 2016). The left, less complex tree shows only the topology of the groups. The respective right tree displays the calculated branch lengths but the receptor groups are condensed into proportional triangles except of ADGRL, ADGRE and the muscarinic acetylcholine receptors (mAChR). The branch point of ADGRL and ADGRE is marked with a red circle. The mAChR branch is marked with a blue circle. Species are: hs, *Homo sapiens* (Mammalia); mm, *Mus musculus* (Mammalia); gg, *Gallus gallus* (Avea); dr, *Danio rerio* (Osteichthyes); sk, *Saccoglossus kowalevskii* (Hemichordata); ci, *Ciona intestinalis* (Tunicata); ac, *Acanthaster planc* (Echinodermata); sp, *Strongylocentrotus purpuratus* (Echinodermata); my, *Mizuhopecten yessoensis* (Mollusca); cg, *Crassostrea gigas* (Mollusca); ob, *Octopus bimaculoides* (Mollusca); dm, *Drosophila melanogaster* (Insecta); la, *Lingula anatina* (Brachiopoda); ct, *Capitella teleta* (Annelida); ce, *Caenorhabditis elegans* (Nematoda); hd, *Hypsibius dujardini* (Tardigrada); rv, *Ramazzottius varieornatus* (Tardigrada); of, *Orbicella faveolata* (Cnidaria); pd, *Pocillopora damicornis* (Cnidaria); aq, *Amphimedon queenslandica* (Parazoa).

In the genomes of the roundworm *C. elegans* and the fruit fly *D. melanogaster*, two and one Latrophilin genes, respectively, have been assigned based on sequence similarities in the N-terminus and the 7TM domain. Re-evaluation of the already described phylogenetic relationship of these invertebrate and vertebrate Adhesion GPCRs (Schioth et al., 2010) revealed a more complex picture placing the 7TM domains of the *C. elegans* Latrophilins LAT-1 and LAT-2 basal to both, the vertebrate Latrophilin (ADGRL) and EMR (ADGRE) groups (Figure 3). In tunicates and evolutionarily old chordates such as lancelet (*Branchiostoma belcheri*) there are obviously no orthologs or paralogs of the ADGRE group, which contains EMR1-4 (ADGRE1-4) and CD97 (ADGRE5) (Figure 3). However, as these can be found in fishes, one can assume that the ADGRE group evolved from the ADGRL group [containing besides LPHN1-3 also ELTD1 (ADGRL4)] in early vertebrate evolution or, alternatively, but more unlikely, was eliminated from all invertebrates. Therefore, the 7TM of LAT-1 and LAT-2 from *C. elegans* and other invertebrates are not in one-to-one orthology to vertebrate Latrophilins but rather share phylogenetic relation to all members of both groups including Latrophilins, ELTD, EMRs, and CD97. Further, based on substitution rates, *C. elegans* *lat-1* and *lat-2* and most invertebrate Latrophilin-like sequences are even more distantly related to the vertebrate Adhesion GPCR groups ADGRL and ADGRE than the *C. elegans* muscarinic acetylcholine receptors *gar-1*/*-2*/*-3* to their vertebrate orthologs/paralogs (Figure 3). Most interestingly, the fruit fly Latrophilin dCirl is even more distantly related to the ADGRL group being placed closer to the Latrophilin-like sequences of *Cnidaria* and *Parazoa* and other Adhesion GPCR groups (Figure 3). Phylogenetic relation built on the basis of the 7TM sequences provides only weak support considering dCirl a member of the Latrophilin group at all. Even if the extracellular N-terminus and its modular composition presents with some structural features of the Latrophilin group, the very distant relation of the 7TM domain may explain differences in their G protein-mediated signal transduction in different species (Lelianova et al., 1997; Müller et al., 2015; Scholz et al., 2017; Nazarko et al., 2018). It has to be noted that already the five vertebrate muscarinic acetylcholine receptors (represented in the lilac triangle in Figure 3) differ in their signaling properties by coupling to G_q/11_ (mAChR-1, -3, -5) and G_i/o_ (mAChR-2, -4).

### The Postulated Teneurin-Latrophilin Interaction Sites Are Not Evolutionarily Old

**FIGURE 4:**
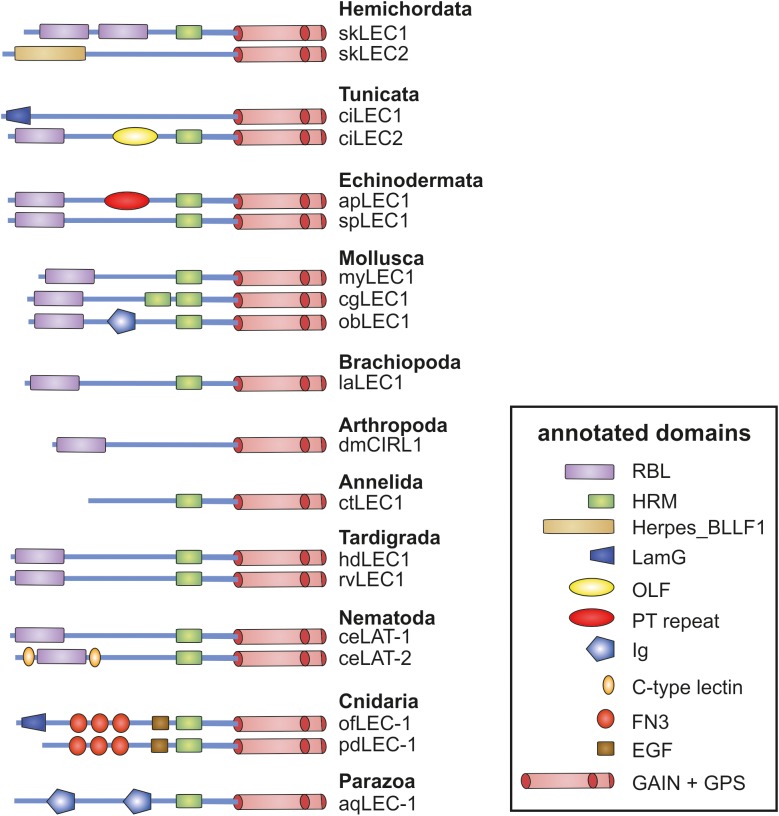
Domain assembly of invertebrate Latrophilins. The N-terminus domain composition of invertebrate Latrophilin-like sequences are shown. Putative conserved domains have been detected with the algorism implemented in NCBI BLAST (Marchler-Bauer et al., 2017). Domain names are given in the box. Note that most of the Latrophilin-like sequences are predicted from genome assemblies, which may contain errors, and are not supported by mRNA data. Species are: sk, *Saccoglossus kowalevskii* (Hemichordata); ci, *Ciona intestinalis* (Tunicata); ac, *Acanthaster planc* (Echinodermata); sp, *Strongylocentrotus purpuratus* (Echinodermata); my, *Mizuhopecten yessoensis* (Mollusca); cg, *Crassostrea gigas* (Mollusca); ob, *Octopus bimaculoides* (Mollusca); dm, *Drosophila melanogaster* (Insecta); la, *Lingula anatina* (Brachiopoda); ct, *Capitella teleta* (Annelida); ce, *Caenorhabditis elegans* (Nematoda); hd, *Hypsibius dujardini* (Tardigrada); rv, *Ramazzottius varieornatus* (Tardigrada); of, *Orbicella faveolata* (Cnidaria); pd, *Pocillopora damicornis* (Cnidaria); aq, *Amphimedon queenslandica* (Parazoa).

Although the phylogenetic analyses on Latrophilins argue at least against the receptor binding to Teneurin and eliciting a conserved signal into the cells, it is still conceivable that an interaction between invertebrate Latrophilins and Teneurins occurs with Latrophilins acting as ligands for Teneurins. The interaction of Latrophilins and Teneurins is mediated by their N-termini and, taking this thought further, one can hypothesize that the 7TM is only modularly attached mediating the appropriated intracellular signal in the different species. As already seen in Figure 1, the worm LAT-1/LAT-2 and the fruit fly dCirl N-termini do not contain an OLF domain and additionally, the HRM domain is missing in dCirl. Detailed analysis of the Latrophilin N-termini in currently available genomes revealed that the ensemble of RBL-, OLF-, and HRM domains in the N-termini of Adhesion GPCRs is found in tunicates (e.g., *Ciona intestinalis*) (Figure 4), in lancelet (*Branchiostoma belcheri*), and Chondrichthyes (*Callorhinchus milii*). In Hemichordata, Echinodermata, Mollusca, Nematoda, Arthropoda, Tardigrada, and Brachiopoda only the GAIN, RBL, and HRM domain (sometimes degenerated or absent) are mostly present (Figure 4), but none of these sequences contains an OLF domain. Interestingly, several invertebrate Latrophilins contain domains (e.g., EGF, Ig, LamG, and FN3) not seen in vertebrate Latrophilins (Figure 4), indicating a modular structure of these Adhesion GPCRs.

**FIGURE 5:**
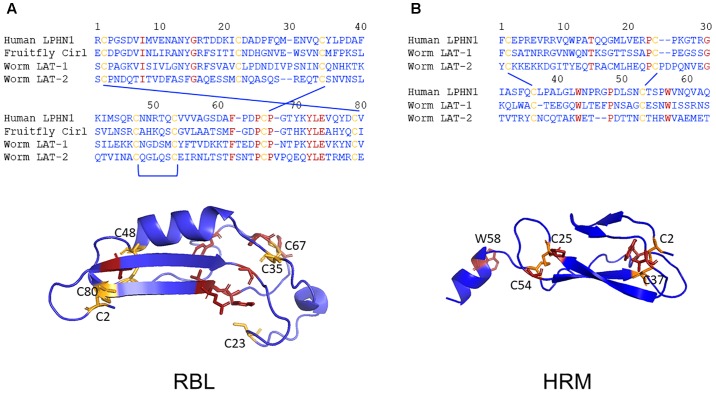
Low evolutionary conservation of the RBL and HMR domains. The amino acid sequence alignments of the putative RBL **(A)** and the HRM **(B)** domains of human LPHN1, fruit fly dCirl and *C. elegans* LAT-1 and LAT-2 are shown. The conserved cysteine (yellow) and other (red) residues are highlighted. Homology models of the three-dimensional structures of the human LPHN1 RBL and HRM domains were generated using Phyre2 (Kelley et al., 2015) based on the best matching templates pdb: c5afbA and pdb: c4dlqA, respectively. Again, the conserved cysteine (yellow) and other (red) residues are highlighted.

Analyses on Latrophilin-Teneurin interactions provide strong evidence that the main site of interaction is the RBL domain with contribution of a short sequence C-terminal of the domain (Figure 2A; Silva et al., 2011; Boucard et al., 2014). Although protein domain identification tools constantly assign RBL and HRM domains in Latrophilins, the amino acid sequence conservation is low (Figure 5). The domain assignment is mainly based on conserved disulfide bond-forming cysteine residues keeping constant folds of the domains. The few other conserved residues mainly surround the conserved disulfide bonds (Figure 5). This suggests that these backbone structures provide the three-dimensional scaffold of the RBL and HRM domains. The remaining amino acid residues most probably participate in specific functions of the two domains. One can speculate that these domains mediate low affinity interactions to proteins or compound or that the sequence variability is the result of a co-evolutionary process with an also variable interaction partner. Although it cannot be fully excluded that invertebrate Latrophilins interact with Teneurins, it does not appear to be likely based on the re-evaluation of existing data above.

## Conclusion and Future Perspectives

Synapse formation is a highly complex and tightly regulated process and although several aspects have been already well understood, many details are still obscure. Latrophilins and Teneurins are both transmembrane proteins which have been described to have implications in synaptogenesis and synapse function. While for Teneurins this has been shown in vertebrate and invertebrate systems, a lot of information is still lacking for Latrophilins. However, a transsynaptic interaction of the two is essential for adhesion and synapse formation in mammals. The question of whether this interaction represents a common principle in the generation of synapses throughout phyla remains unanswered, mainly due to lacking experimental evidence, but is highly intriguing. Similarities in expression, seemingly functional redundancy in *Drosophila* and a general evolutionary conservation makes it tempting to conclude that this transsynaptic interaction is old and also meaningful in invertebrate species. However, a closer look at phylogenetic evidence and existing data sheds light on a different picture.

Our phylogenetic analyses indicate that, although basal metazoans already contain Adhesion GPCR, clearly Latrophilin-7TM-related sequences only appeared at the level of Eumetazoa and thus, later in evolution than Teneurins. The connected N-termini contain RBL- and HRM-like domains but not constantly. Further, the OLF domain only appears in the N-termini of Latrophilins in early chordate evolution. Although the conserved cysteine bonds and a few other conserved positions allow for assignments as RBL and HRM domains, most of the remaining sequence is highly variable in these domains. This indicates that the RBL and HRM domains in Latrophilins may have specific functions in the different species and/or underwent co-evolution with interaction partners rather than mediating evolutionarily conserved protein-protein interactions. This analysis yields some evidence that a conserved interaction of Latrophilins and Teneurins in invertebrates might not be likely. It cannot be excluded that additional, not yet identified interaction sites in Latrophilins exist, which represent highly conserved sequences. Further, the role of other proteins or molecules aiding or promoting the interaction cannot be evaluated. For instance, dystroglycans have been discussed to be part of a larger complex (Woelfle et al., 2015). However, if a physical interaction may occur, a potential signal elicited by the Adhesion GPCR is not comparable to signals transduced by mammalian Latrophilins as invertebrate Latrophilins, in particular the homolog in *Drosophila*, are not one-to-one homologs of mammalian Latrophilins, but also bear resemblance to other Adhesion GPCRs. This argument is further supported by experimental data highlighting distinct signaling cascades activated by Latrophilin homologs of different species: While mammalian LPHN1 can signal via G_s_ or G_i_ proteins (Müller et al., 2015; Nazarko et al., 2018), *Drosophila* dCirl activates G_i_ proteins and *C. elegans* LAT-1 G_s_ proteins. A functional evaluation of these different cascades will shed light on the impact of these cascades.

We cannot exclude an interaction between Latrophilins and Teneurins in invertebrates, however, the mode of interaction might be realized differently from their mammalian counterparts. While both groups of proteins have essential functions in invertebrates and the ones of Teneurins in particular are highly conserved roles across phyla, they might not realize this role via the help of Latrophilins. Invertebrates have less complex regulatory circuits and hence, different requirements for synapse formation and function. Thus, it would not be surprising that they utilize different mechanisms to establish and maintain synapses and their function.

Future analyses need to focus on gaining a better understanding of the physiological functions mediated by both, Latrophilins and Teneurins, in mammals and invertebrates. These will help understand similarities as well as differences in the function of each receptor in different contexts and aid the understanding of the molecular mechanisms underlying synaptogenesis and neuronal wiring in vertebrates and invertebrates. It will be highly interesting to gain information on the existence and composition of potential synaptic complexes involving Latrophilins and/or Teneurins. Further, identifying interaction interfaces of mammalian Latrophilins with Teneurins can be highly informative for the prediction and characterization of a potential interaction in other species.

## Author Contributions

TS and SP researched and wrote the manuscript.

## Conflict of Interest Statement

The authors declare that the research was conducted in the absence of any commercial or financial relationships that could be construed as a potential conflict of interest.
